# Case Report: a family presenting with β-adrenergic/vasopressin-responsive bilateral macronodular adrenal disease with an *ARMC5* mutation treated with metyrapone monotherapy for more than 5 years

**DOI:** 10.3389/fendo.2026.1852080

**Published:** 2026-07-21

**Authors:** Masanori Arai, Masato Ono, Ryota Inoue, Kazuki Tajima, Kota Aomori, Tomoyuki Tatenuma, Sawako Suzuki, Shoji Yamanaka, Satoshi Fujii, Yasuo Terauchi, Jun Shirakawa

**Affiliations:** 1 Department of Endocrinology and Metabolism, Graduate School of Medicine, Yokohama City University, Yokohama, Japan; 2 Laboratory of Diabetes and Metabolic Disorders, Institute for Molecular and Cellular Regulation (IMCR), Gunma University, Maebashi, Japan; 3 Department of Urology, Graduate School of Medicine, Yokohama City University, Yokohama, Japan; 4 Department of Endocrinology, Hematology and Gerontology, Graduate School of Medicine, Chiba University, Chiba, Japan; 5 Department of Pathology, Yokohama City University Hospital, Yokohama, Japan; 6 Department of Molecular Pathology, Yokohama City University Graduate School of Medicine, Yokohama, Japan

**Keywords:** *ARMC5*, bilateral macronodular adrenal disease, BMAD, Cushing’ s syndrome, Japanese, metyrapone

## Abstract

Bilateral macronodular adrenal disease (BMAD), also referred to as primary bilateral macronodular adrenal hyperplasia (PBMAH), represents a rare etiology of subclinical or overt Cushing’s syndrome (CS), characterized by the presence of multiple large nodules in both adrenal glands. Although historically considered a sporadic condition, familial cases of BMAD have become increasingly recognized in recent years. As early surgical intervention is typically chosen for these familial cases, evidence on the long-term effectiveness of conservative medical therapy remains scarce. Here, we report a Japanese parent–child pair with subclinical and overt CS with genetically confirmed BMAD in which both individuals harbored *ARMC5* mutations and exhibited aberrant hormone responses and were managed with long-term metyrapone monotherapy. In both cases, sustained biochemical control of adrenocortical hyperfunction was achieved in more than 5 years. One patient developed progressive hypokalemia, eventually leading to unilateral adrenalectomy. These findings suggest that metyrapone monotherapy serves as an effective noninvasive treatment strategy for select familial BMAD patients, although close monitoring is warranted for potential complications.

## Introduction

Bilateral macronodular adrenal disease (BMAD), also referred to as ACTH-independent macronodular adrenal hyperplasia (AIMAH) or primary bilateral macronodular adrenal hyperplasia (PBMAH), is a rare etiology of subclinical or overt Cushing’s syndrome (CS), accounting for <2% of endogenous CS cases. BMAD produces excessive amounts of cortisol through a distinctive mechanism involving aberrant adrenal expression of hormone receptors that respond to various ligands, including catecholamines, vasopressin, gastric inhibitory peptide (GIP), angiotensin II (AII), luteinizing hormone (LH), follicle-stimulating hormone (FSH), thyrotropin-releasing hormone (TRH), and 5-hydroxytryptamine receptor 4 (5-HT4), as well as pituitary-derived adrenocorticotrophic hormone (ACTH) (1, 2). One proposed mechanism is that aberrant receptor expression may promote paracrine ACTH production within the adrenal cortex, contributing to autonomous cortisol secretion and subsequent hypercortisolemia (3).

BMAD was previously considered a predominantly sporadic disorder; however, accumulating evidence in recent years has revealed several familial lineages (3–11). Genetic analyses have implicated multiple susceptibility genes in the pathogenesis of BMAD, including armadillo-repeat containing 5 (*ARMC5*), adenomatous polyposis coli (*APC*), fumarate hydratase (*FH*), melanocortin type 2 receptor (*MC2R*), guanine nucleotide-binding protein, alpha-stimulating activity polypeptide 1 (*GNAS*), multiple endocrine neoplasm type 1 (*MEN1*), protein kinase A catalytic subunit alpha (*PRKACA*), phosphodiesterases (PDEs) such as *PDE11A* and *PDE8B*, and lysine demethylase 1A (*KDM1A*) (10–15). Among these, mutations in *ARMC5* are the most prevalent and are detected in approximately 80% of familial BMAD cases, indicating their pathogenic significance (2, 12, 15).

Surgical adrenalectomy remains a standard therapeutic option for BMAD patients who present with overt CS when applicable. Although bilateral adrenalectomy was historically regarded as the standard surgical approach, unilateral adrenalectomy targeting the dominant adrenal gland has increasingly been considered in selected patients, particularly those with mild autonomous cortisol secretion. Nonsurgical treatment can be considered for individuals with mild cortisol excess or those who are ineligible or unwilling to undergo surgery. Available pharmacologic options for CS include ketoconazole, metyrapone, mitotane, pasireotide, osilodrostat, and dopamine agonists; however, drug availability and clinical accessibility vary considerably among countries. Among these agents, metyrapone remains one of the most widely used steroidogenesis inhibitors in Japan. Despite its clinical application, the long-term effectiveness of metyrapone in BMAD, particularly in familial cases, remains poorly characterized (16, 17).

Here, we report a parent–child familial BMAD case in which both individuals harbored *ARMC5* mutations and exhibited aberrant hormone responses and were managed with metyrapone monotherapy for more than 5 years.

## Case presentation

### Patient 1

**Figure 1:**
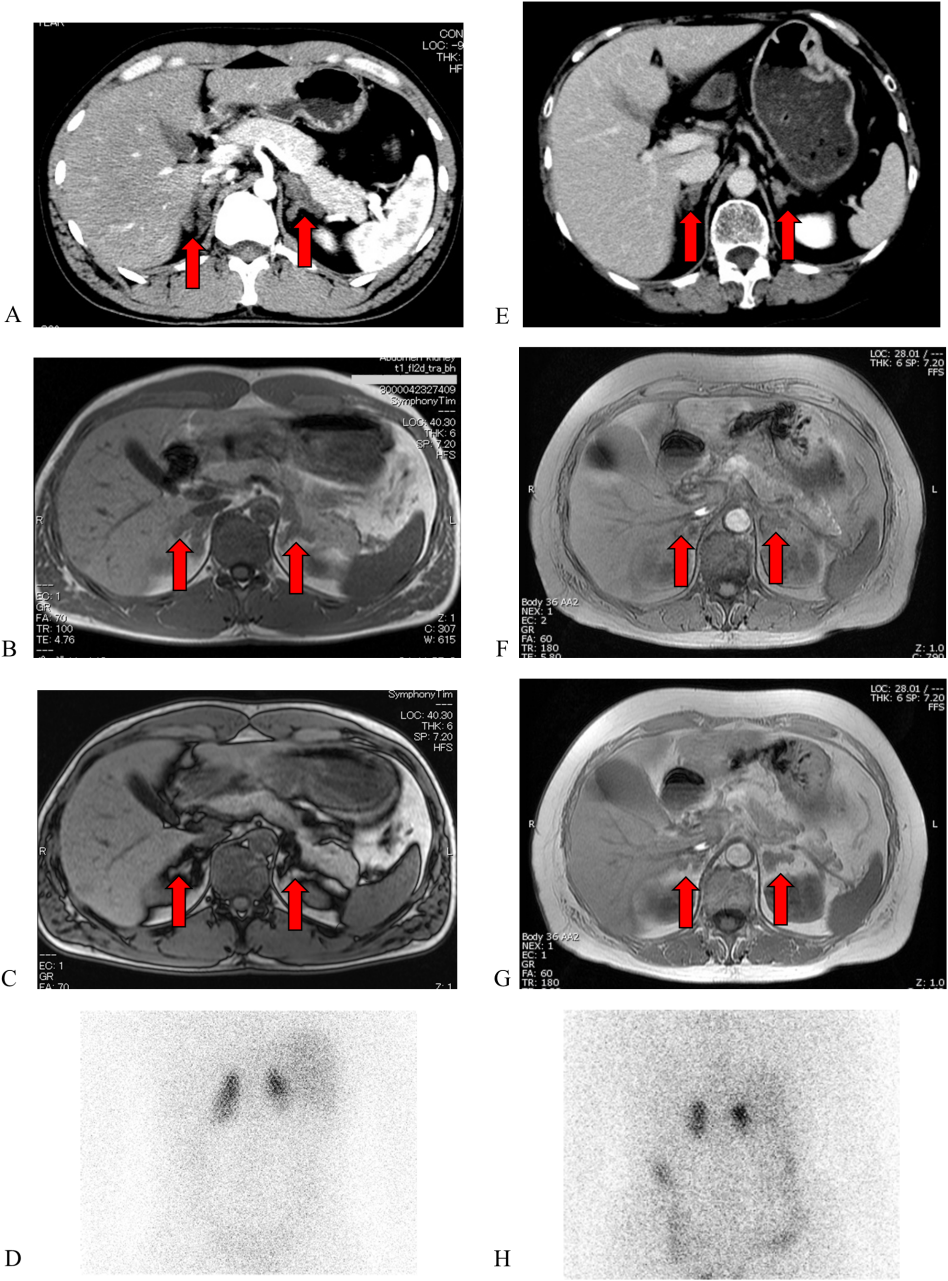
Various imaging findings of the adrenal glands in Patient 1 **(A–D)** and Patient 2 **(E–H)**. **(A, E)** contrast-enhanced CT, **(B, F)** in phase magnetic resonance imaging (MRI), **(C, G)** opposed-phase MRI, **(D, H)** ^131^I-adosterol adrenal scintigraphy (posterior view). Red arrows point to the enlarged adrenal glands.

A 39-year-old Japanese man with no specific complaints was found to have elevated liver enzyme levels during a health check-up. He subsequently consulted a local physician, and an abdominal computed tomography (CT) scan incidentally revealed bilateral adrenal gland enlargement with multiple nodules (**Figure 1A**). He was referred to our department for further evaluation.

**Figure 2:**
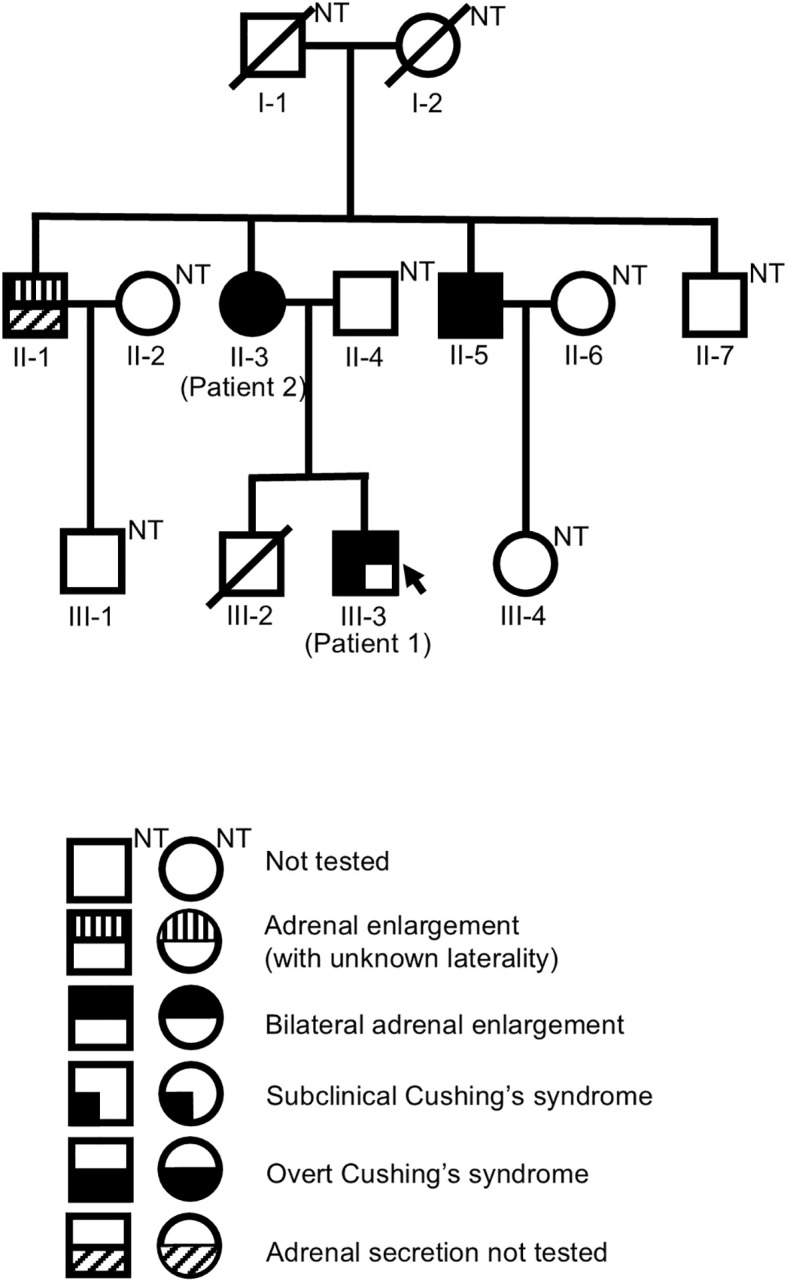
Pedigree of a Japanese family with BMAD, including Patient 1 (III-3) and Patient 2 (II-3). The black arrow indicates the proband.

The patient had a prior diagnosis of anxiety neurosis and had been taking clotiazepam intermittently. He reported no use of prescribed or illicit drugs, apart from two types of nutritional supplements. There was no known family history of CS on the paternal side. However, his maternal family history was notable for adrenal abnormalities: one maternal uncle had previously been diagnosed with BMAD and was receiving metyrapone therapy at another hospital, although detailed records were unavailable. Another maternal uncle had documented bilateral adrenal enlargement but declined further diagnostic assessment. His mother (Patient 2) had a history of hypertension and osteoporosis and was subsequently diagnosed with BMAD. His maternal grandparents had not undergone adrenal evaluation, although his maternal grandmother was reported to be obese. A detailed pedigree chart is shown in **Figure 2**.

At the initial evaluation, his height was 161 cm and weight was 59 kg (body mass index 22.7 kg/m^2^), and physical examination revealed no apparent signs suggesting CS, such as moon face, buffalo hump, central obesity, striae rubrae, acne, skin atrophy, or hirsutism. His blood pressure and pulse rate were within normal ranges. Bone densitometry (dual-energy X-ray absorptiometry; DXA) demonstrated slightly decreased bone mineral density of the lumbar vertebrae (0.888 g/cm^2^, T score -1.3) and femoral neck (0.631 g/cm^2^, T score -1.8). Laboratory examination revealed a loss of normal diurnal variation in serum cortisol levels and suppression of plasma ACTH and dehydroepiandrosterone sulfate (DHEA-S) levels (**Table 1**). Other baseline endocrine and biochemical parameters, including liver enzyme levels, were within normal ranges. Magnetic resonance imaging (MRI) revealed multiple isointense nodules on T1-weighted images (**Figures 1B, C**), with relative hypointensity on opposed-phase imaging compared with in-phase imaging (**Figures 1C** **vs.** **1B**), which is consistent with lipid-rich adrenal lesions. Endocrine dynamic tests revealed a lack of cortisol suppression in response to both overnight 1 mg and 8 mg dexamethasone suppression tests (DSTs). Cortisol and ACTH responses to corticotropin-releasing hormone (CRH) stimulation were blunted, whereas cortisol and aldosterone levels exhibited exaggerated responses to short-duration synthetic ACTH stimulation. ^131^I-adosterol scintigraphy demonstrated bilateral tracer uptake in the adrenal glands, with predominant accumulation in the left adrenal gland (**Figure 1D****).** In summary, the patient fulfilled the diagnostic criteria for subclinical CS (SCS) commonly used in Japan at that time including the presence of adrenal incidentalomas, the absence of physical features typical of CS, normal basal cortisol levels, autonomous cortisol secretion confirmed by both 1-mg and 8-mg overnight dexamethasone suppression tests, and unilateral predominant uptake with contralateral suppression on adrenal scintigraphy (18). The patient was therefore diagnosed with SCS due to BMAD. In addition, the patient also fulfilled the criteria for mild autonomous cortisol secretion in 2023 European Society of Endocrinology Clinical Practice Guidelines developed in collaboration with the European Network for the Study of Adrenal Tumors (ENSAT) (19). **Supplementary Table 1** summarizes the endocrine evaluations of both patients, including basal ACTH and cortisol levels, dexamethasone suppression tests, CRH stimulation tests, synthetic ACTH stimulation tests, urinary free cortisol levels, renin-aldosterone measurements, assay methods, and corresponding normal reference ranges.

**Table 1:** Baseline laboratory data.

Parameters	Patient 1	Patient 2
Fasting plasma glucose (mg/dL)	97	106
HbA1c (%)	6.2	6.3
White blood cell count (/μL)	4700	6500
Neutrophils (%)	69.9	76.6
Eosinophils (%)	1.5	1.7
Basophils (%)	0.7	0.5
Monocytes (%)	9.8	8.0
Lymphocytes (%)	18.1	13.2
Aspartate aminotransferase (U/L)	20	23
Alanine aminotransferase (U/L)	23	18
γ-glutamyl transferase (U/L)	16	25
Serum sodium (mmol/L)	139	142
Serum potassium (mmol/L)	3.9	4.3
Triglycerides (mg/dL)	49	80
High density lipoprotein cholesterol (mg/dL)	71	57
Low density lipoprotein cholesterol (mg/dL)	76	107

To evaluate the potential involvement of ectopic hormone receptor expression, a series of stimulation tests was conducted. The percentage increase in peak serum cortisol from baseline was 56.4% in response to the upright posture, no response to desmopressin, 38.7% in response to vasopressin following 8 mg dexamethasone suppression (20), 15.1% with luteinizing hormone-releasing hormone (LHRH), 9.3% with thyrotropin-releasing hormone (TRH), 2.5% with metoclopramide, 50.3% with 75 g oral glucose, and 26.3% with 25 g intravenous glucose. Cortisol response to stimulation was classified as follows: ≥50% increase indicated a positive response; 25%–50%, a partial response; and <25%, no response (1). On the basis of these criteria, upright posture, vasopressin (Pitressin) stimulation, and oral glucose administration induced a significant cortisol response, indicating the presence of aberrant receptor expression.

**Figure 3:**
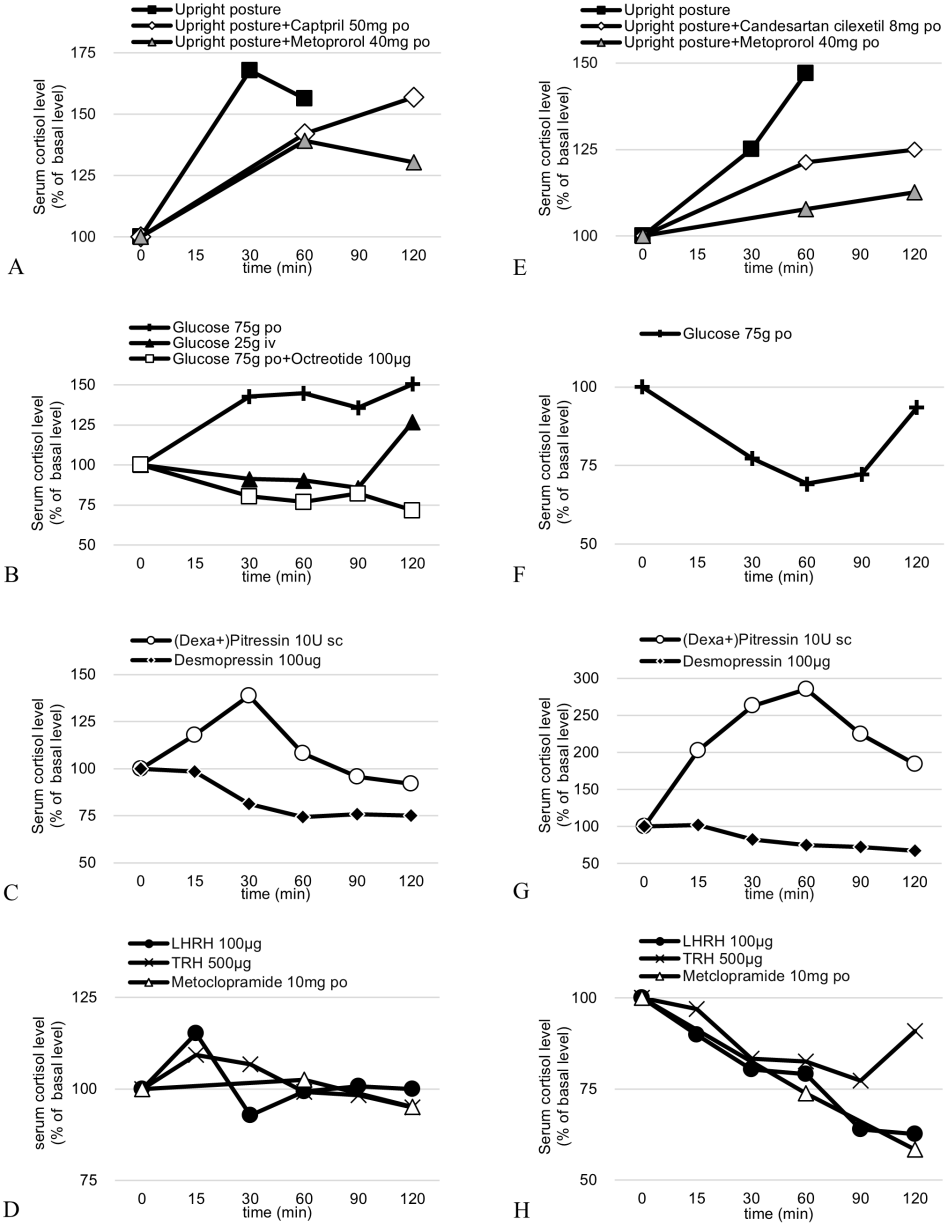
Serum cortisol levels in response to various stimulation tests in Patient 1 **(A–D)** and Patient 2 **(E–H)**. **(A, E)** Upright posture; **(B, F)** 75 g oral glucose tolerance test (OGTT); **(C, G)** Vasopressin receptor agonist stimulation test; and **(D, H)** other stimulation tests.

Combination tests further demonstrated that cortisol levels increased by 57.9% with the upright posture plus oral captopril (50 mg) and by 39.1% with the upright posture plus oral metoprolol (40 mg), whereas no response was observed with the combination of oral glucose (75 g) and subcutaneous octreotide (100 μg). The time course data of serum cortisol levels during these stimulation tests are shown in **Figure 3**. These results suggested that inappropriate expression of receptors responsive to vasopressin, adrenaline, and GIP may have contributed to the autonomous production of cortisol in this case. Sanger sequencing of peripheral blood samples revealed a heterozygous p.R593W (c.1777C>T) mutation in the *ARMC5* gene (**Supplementary Figure 1**).

Although early surgical intervention (left adrenalectomy) was recommended, the patient declined and opted for pharmacologic management. Because autonomous cortisol excess was relatively moderate, treatment with metyrapone was initiated at a dosage of 500 mg/day during hospitalization, 7 months after diagnosis. Urinary free cortisol level rapidly decreased to 35.7 μg/day following treatment initiation and remained within the normal range during follow-up. No clinical findings suggestive of adrenal insufficiency were observed during long-term administration. Persistent suppression of plasma ACTH necessitated gradual dosage escalation to 1000 mg/day by 24 months and further to 3000 mg/day by 63 months after treatment initiation. The patient’s prescribed medications for subclinical CS and for comorbidities associated with hypercortisolemia over the treatment course are shown in **Supplementary Figure 2A**. Throughout treatment, no overt symptoms associated with hypercortisolism were observed, and HbA1c remained stable; however, progressive dyslipidemia was noted, with LDL-C peaking at 141.4 mg/dL at the most recent visit. At 18 months post-diagnosis, bisphosphonate therapy was initiated for osteopenia (lumbar and femoral neck T scores of -1.3 and -1.6, respectively). Bone mineral density trends are shown in **Supplementary Figure 2B**. The patient also received antihypertensive therapy with amlodipine besilate, atenolol, and telmisartan.

Serial thin-slice CT revealed a gradual increase in adrenal gland volume, peaking at 42 months, followed by slight regression (**Supplementary Figure 2C**). Urinary free cortisol normalized to creatinine remained between 30 and 100 μg/gCr during follow-up. Plasma ACTH and cortisol levels were suppressed during the first 40 months of metyrapone therapy but subsequently fluctuated.

At 47 months, the patient was diagnosed with localization-related epilepsy, presumably unrelated to adrenal pathology or treatment and was started on antiepileptic medication. During the early treatment period, overt manifestations attributable to hypercortisolism were not apparent, and HbA1c remained relatively stable. However, approximately 60 months after diagnosis, progressive hypertension and worsening dyslipidemia became evident, suggesting transition toward overt CS. At that time, unilateral adrenalectomy of the dominant adrenal gland was again recommended; however, the patient preferred continuation of metyrapone monotherapy. Over the 69-month period, body weight increased by 2 kg and remained stable thereafter. Despite maintaining basal ACTH and cortisol levels within normal limits and preventing overt Cushing’s complications, the patient developed progressive hypokalemia (nadir: 2.9 mEq/L), prompting the addition of oral potassium aspartate (300 mg/day) at 84 months. This hypokalemia was attributed to excess 11-deoxycorticosterone, a consequence of persistent ACTH stimulation and metyrapone-mediated 11β-hydroxylase inhibition. From 72 months post-diagnosis, the patient reported mild tingling sensation in his stomach occurring several hours after taking metyrapone, noting that the symptom emerged only after the dose was increased.

Given the refractory hypokalemia and above-mentioned adverse event, surgical intervention was reconsidered and subsequently performed. A laparoscopic left adrenalectomy was completed at 89 months post-diagnosis. Perioperative hydrocortisone sodium succinate (100 mg every 8 hours) was administered and tapered by postoperative day (POD) 5. Metyrapone therapy was temporarily suspended on the day of surgery and restarted on POD6 at 2500 mg/day, after which it was subsequently reduced to 2000 mg/day by discharge (POD8). The excised left adrenal sample weighed 57 g and showed numerous nodules (**Supplementary Figures 4A, B**). Histological examination revealed diffuse cortical hyperplasia without definitive neoplastic features (**Supplementary Figures 4C, D**).

After unilateral adrenalectomy, the metyrapone dosage was reduced from 1750 mg/day to 1500 mg/day, and oral potassium supplementation was discontinued because hypokalemia had resolved. On the other hand, compensatory hypertrophy of the right adrenal gland was observed, with the volume increasing from 18.4 cm³ to 26.2 cm^3^ over 27 months, accompanied by worsening hypertension. Consequently, antihypertensive drugs were intensified with increased doses of telmisartan (80 mg/day), amlodipine (10 mg/day), and carvedilol (10 mg/day) at 20 months post-surgery.

### Patient 2

A 68-year-old Japanese woman, the mother of Patient 1, presented for evaluation of adrenal function following the recent diagnosis of BMAD in both her younger brother and her son. At that time, only a nonresting serum cortisol level was measured, which revealed a mildly elevated level of 14.8 μg/dL. A CT scan performed subsequently at an outside facility revealed bilateral adrenal enlargement with multiple macronodular structures (**Figure 1E**). She was then referred to our department for further endocrinological assessment.

Contrast-enhanced MRI of the adrenal glands revealed multiple isointense nodules on both T1- and T2-weighted images, with no signal suppression on opposed-phase imaging (**Figures 1F, G**). Her medical history included dyslipidemia for 15 years, hypertension for 5 years, and osteoporosis 2 years earlier. She had no history of fragility fractures. Her obstetric history included two pregnancies and two deliveries, without notable perinatal complications including macrosomia. Her current medications included fluvastatin (20 mg/day), candesartan cilexetil (8 mg/day), amlodipine besilate (2.5 mg/day), alfacalcidol (1 μg/day), raloxifene hydrochloride (60 mg/day), and lafutidine (20 mg/day). She also had a significant family history of BMAD, and her older child had died at age 27 from dilated cardiomyopathy (**Figure 2**).

On admission, her height was 144 cm and weight was 52 kg (BMI: 25.0 kg/m^2^), which met the diagnostic criteria for obesity according to the Japan Society for the Study of Obesity (21). Physical examination revealed characteristic features of hypercortisolism, including moon face, central obesity, striae rubrae, and hirsutism in the lower limbs. Her blood pressure was 134/75 mmHg and heart rate was 96 bpm under antihypertensive therapy. Contrast-enhanced pituitary MR image revealed an empty sella. DXA demonstrated significant osteoporosis: despite ongoing anti-osteoporotic treatment, lumbar spine BMD was 0.602 g/cm² (T score of -3.4), and the femoral neck BMD of 0.495 g/cm^2^ on the right and 0.500 g/cm^2^ on the left (T scores of -2.6 and -2.6, respectively).

Laboratory examination revealed a loss of diurnal variation in serum cortisol levels, suppressed plasma ACTH and DHEA-S levels, and a mildly elevated HbA1c concentration of 6.3%. Other blood tests, including those for pituitary hormones, were within normal ranges (**Table 1**). Hormonal stimulation tests demonstrated a lack of cortisol suppression with overnight 1 mg and 8 mg DST. Cortisol and ACTH responses to CRH stimulation were preserved, whereas synthetic ACTH (short duration) elicited exaggerated cortisol and aldosterone responses. ^131^I-adosterol scintigraphy revealed bilateral adrenal uptake with a right-sided predominance (**Figure 1H**). These findings, despite preserved ACTH and cortisol responsiveness to CRH, were consistent with BMAD associated with ACTH-independent adrenal CS.

Hormone stimulation tests revealed a 46.9% increase in serum cortisol in response to upright posture and a 185.4% increase with vasopressin after 8 mg dexamethasone pretreatment (20). No significant cortisol responses were observed with desmopressin (1.9%), LHRH, TRH, metoclopramide, or 75 g oral glucose (**Figure 3**). These results suggested aberrant expression of vasopressin V1 receptors and β-adrenergic receptors. A 25.0% increase in cortisol concentration was observed with upright posture combined with candesartan cilexetil (8 mg), and a 12.6% increase was observed with metoprolol (40 mg) (**Figure 3**), supporting aberrant receptor-mediated cortisol production.

Sanger sequencing identified the same heterozygous *ARMC5* variant (c.1777C>T, p.R593W) in Patient 2 as previously detected in her son (Patient 1). Additional analyses of other genes associated with BMAD were not performed because comprehensive BMAD gene panels were not available in our institution at the time of evaluation. Although adrenalectomy was proposed as a first-line treatment, medical management was pursued. Metyrapone therapy was initiated at 500 mg/day during hospitalization three months after diagnosis. Urinary free cortisol level promptly decreased to 35.9 μg/day after treatment initiation, and biochemical control was maintained thereafter without evidence of adrenal insufficiency or other major adverse effects. The same dosage of metyrapone was continued throughout follow-up.

The prescribed medications for CS and hypercortisolemia-related comorbidities over the clinical course are shown in **Supplementary Figure 3A**. Throughout the follow-up period, patient’s lipid levels (LDL-cholesterol and total cholesterol) remained well controlled with statins. HbA1c levels gradually increased, leading to a diagnosis of diabetes 32 months after the initial CT; however, glycemic control remained adequate (HbA1c < 7%) with lifestyle modification alone. Bone mineral density remained stable under weekly bisphosphonate therapy (**Supplementary Figure 3B**). Adrenal volume did not significantly increase, and the nonresting serum cortisol concentration and creatinine-adjusted urinary free cortisol concentration remained within a modestly elevated range (**Supplementary Figure 3C**). Both plasma ACTH levels and serum cortisol levels were suppressed during the initial 30 months of metyrapone therapy but gradually increased thereafter. ACTH levels surpassed 10 pg/mL in most subsequent measurements after 80 months. During the 65-month follow-up period, the patient experienced a modest weight gain of 3 kg, which subsequently stabilized. Blood pressure control remained suboptimal, necessitating gradual escalation of antihypertensive therapy with calcium channel blockers and angiotensin receptor blockers.

## Discussion

We report two familial BMAD cases harboring an identical pathogenic variant in the *ARMC5* gene, each exhibiting aberrant β-adrenergic and vasopressin receptor-mediated cortisol secretion. Both patients were managed with metyrapone monotherapy for more than 5 years: 83.4 months in Patient 1 and more than 110 months to date in Patient 2. BMAD itself represents a rare etiology of CS, and cases treated exclusively with long-term medical therapy are exceedingly uncommon. To our knowledge, this is the first report to detail the sustained efficacy and longitudinal outcomes of medical therapy in familial BMAD over such prolonged durations, as prior case series and reports have typically involved therapeutic courses lasting less than one year (16, 17, 22).

Despite sharing key clinical and genetic features, the disease trajectories of these two patients exhibited notable differences. In one case, metyrapone monotherapy achieved durable control of hypercortisolemia and adrenal morphology. In the other case, progressive adrenal enlargement necessitated escalation of the metyrapone dosage, eventually leading to hypokalemia and the need for unilateral adrenalectomy for symptom control. These divergent outcomes highlight the interindividual variability in disease expression, even among familial cases carrying the same *ARMC5* mutation.

Recent advances in steroid metabolomics have further refined our understanding of BMAD subtypes. A mass spectrometry-based steroid profiling study demonstrated marked biochemical distinctions among BMAD with *ARMC5* mutations, BMAD without such mutations, and other forms of adrenal CS (ACS). Specifically, levels of 11-deoxycortisol, corticosterone, 11-deoxycorticosterone, and 18-hydroxycortisol differed significantly across these groups. This study also suggested a gradation in cortisol production capacity, with the highest levels in ACS, intermediate levels in BMAD without *ARMC5* mutations, and the lowest levels in BMAD with *ARMC5* mutations, which was paralleled by an inverse trend in 18-deoxycortisol and corticosterone concentrations (23). These findings support the notion that BMAD patients with shared *ARMC5* mutations may exhibit a distinct steroidogenic signature, which in turn may influence their clinical course and therapeutic responsiveness. Volumetric imaging analyses of BMAD revealed that total adrenal volume is positively correlated with urinary excretion of 17-hydroxycorticosteroids (17-OHCS) rather than with urinary free cortisol (24). In particular, analyses stratified by ethnicity and laterality demonstrated that this correlation was most pronounced between 17-OHCS and left adrenal volume among African American participants (24). As 17-OHCS encompasses a range of corticosteroid metabolites, including cortisol, tetrahydrocortisol, tetrahydrocortisone, allo-tetrahydrocortisol, 6β-hydroxycortisol, cortisone, and tetrahydrodeoxycortisol (THS), it serves as a more integrative marker of total corticosteroid turnover than cortisol alone does (25). Importantly, THS is a primary metabolite of 11-deoxycortisol, suggesting that the observed discrepancies between 17-OHCS and cortisol with respect to adrenal volume may be attributable to differential 11-deoxycortisol production. Previous studies have suggested that inefficient steroidogenesis may contribute to relatively mild hypercortisolemia despite marked adrenal enlargement in BMAD patients (12, 26). Differences in adrenal volume between the parent-child pair in the present study may therefore reflect heterogeneity in steroidogenic profiles, even in individuals sharing the same genetic variant. Since steroidomic analyses were not performed in this study, further investigations are warranted.

Integrating these observations with the aforementioned steroid profiling data, we hypothesized that the disparity in adrenal gland size between our two BMAD patients, despite sharing an identical *ARMC5* variant, may reflect differences in the magnitude of the 11-deoxycortisol pool rather than in the level of cortisol per se. In Patient 1, we speculate that periodic surges in 11β-hydroxylase activity may have transiently amplified cortisol synthesis, overwhelming the suppressive effect of metyrapone and leading to progressive adrenal enlargement. These findings suggest that in BMAD patients with massive adrenal hypertrophy, cortisol production may be more labile, even under steroidogenesis inhibition by metyrapone, necessitating vigilant monitoring and consideration of surgical intervention. With respect to alternative agents that suppress cortisol synthesis, the clinical use of osilodrostat has recently expanded. Like metyrapone, osilodrostat inhibits 11β-hydroxylase; however, it has a higher binding affinity for the enzyme and a longer half-life. Furthermore, it differs from metyrapone in that it also exhibits 18-hydroxylase inhibitory activity (27). These pharmacological distinctions may translate into differential therapeutic efficacy in BMAD. Future studies and longitudinal clinical reports are needed to determine whether long-term osilodrostat administration can achieve sustained disease control in BMAD patients without the need for adrenalectomy, as suggested by the present case series.

The shared *ARMC5* mutation identified in both patients, p.R593W (c.1777C>T), is a previously reported pathogenic variant, with at least 4 cases described across 2 reports (6, 28–30). Clinical data were available for 3 patients, all of whom demonstrated cortisol hyperresponsiveness to vasopressin stimulation, which is consistent with the aberrant receptor expression observed in our patients. Aberrant responses to metoclopramide were noted in one individual. These findings reinforce the hypothesis that *ARMC5* inactivation may result in the overexpression of ectopic G-protein receptors, particularly β-adrenergic and vasopressin V1 receptors (5, 6, 31).

Recent mechanistic studies have further elucidated the functional consequences of ARMC5 loss-of-function. ARMC5 may form an E3 ubiquitin ligase complex with CUL3 and RBX1, specifically targeting RPB1, the largest subunit of RNA polymerase II (Pol II), for ubiquitination (29). *ARMC5* mutations may thereby impair Pol II degradation and disrupt the transcriptional regulation of downstream effector genes, contributing to the pathogenesis of BMAD. Nevertheless, the complete molecular cascade remains incompletely defined, and further studies are needed to clarify the mechanistic links among ARMC5 dysfunction, receptor dysregulation, and adrenal hyperplasia.

In the assessment of aberrant hormone receptor activity using stimulation test, only Patient 1 showed findings suggestive of aberrant GIPR responsiveness. Although KDM1A inactivation has been implicated in many cases of hereditary food-dependent BMAD (10, 11), genetic analyses beyond *ARMC5* were not performed in the present study. This limitation restricts the mechanistic interpretation of the observed receptor phenotype. Furthermore, *ARMC5* is considered a tumor suppressor gene, and BMAD development is thought to occur through a “two-hit” mechanism involving secondary somatic alterations within adrenal nodules. Loss of wild-type ARMC5 function is believed to represent an important initiating event in adrenal tumorigenesis (32). In this context, the number of adrenal nodules may be influenced by the frequency and distribution of second somatic events. In contrast, the mechanisms determining the growth dynamics and functional heterogeneity of individual nodules remain incompletely understood. In the present study, sequencing analysis of excised adrenal tissue could not be performed, and therefore the potential contribution of somatic second-hit alterations could not be determined. In Patient 1, we speculate that intermittent increases in steroidogenic activity may have contributed to progressive enlargement of pre-existing nodules despite metyrapone therapy; however, this hypothesis remains speculative and requires further investigation.

In conclusion, we describe two familial BMAD patients harboring a shared *ARMC5* p.R593W mutation, both of whom exhibited aberrant β-adrenergic and vasopressin V1 receptor-mediated cortisol secretion and were managed successfully with long-term metyrapone monotherapy for more than 5 years. While one patient maintained stable disease control for more than 9 years, the other developed progressive adrenal enlargement and ultimately required unilateral adrenalectomy after 83.4 months of metyrapone monotherapy. These cases illustrate both the potential and limitations of long-term pharmacologic management in BMAD patients, emphasizing the need for individualized therapeutic strategies informed by hormonal profiling and adrenal imaging.

## Data Availability

The raw data supporting the conclusions of this article will be made available by the authors, without undue reservation.

## Supplementary material

Supplementary Figure 1 Sanger sequencing analysis of the ARMC5 gene. **(A)** Patient 1 and **(B)** Patient 2. An identical heterozygous mutation, p.R593W (c.1777C>T), was identified in both cases. Red arrows indicate the nucleotide substitution.

Supplementary Figure 2 Clinical course of Patient 1. **(A)** Clinical timeline and prescribed medications. **(B)** Bone mineral density (BMD) measured by dual-energy X-ray absorptiometry (DXA). **(C)** Adrenal volume estimated by unenhanced thin-slice CT.

Supplementary Figure 3 Clinical course of Patient 2. **(A)** Clinical timeline and prescribed medications. **(B)** Bone mineral density (BMD) measured by dual-energy X-ray absorptiometry (DXA). **(C)** Adrenal volume estimated by unenhanced thin-slice CT.

Supplementary Figure 4 Macroscopic and histopathological findings of the left adrenal gland in Patient 1. **(A)** Gross appearance of the resected left adrenal gland. **(B)** Cut surface of the sample showing multiple nodules. **(C, D)** Hematoxylin and eosin (H&E) staining of the nodule. Scale bar = 500 μm.

Supplementary Table 1 Endocrine evaluations. **(A)** measured by CLEIA; **(b)** measured by CLIA; **(c)** measured by EIA. Normal reference ranges: serum cortisol at 08:00 h (7.07-19.6 μg/dL) and 24:00 h (< 5.0 μg/dL); plasma ACTH (7.2–63.3 pg/mL), DHEA-S (1060–4640 for Patient 1, 120–1330 for Patient 2), urinaly free cortisol (4.3-176 μg/day), plasma renin activity at rest (0.2-2.3 ng/mL/hr), plasma aldosterone concentration at rest (4.0-82.1 pg/dL).
