# Supplementary material for: Case Report: a family presenting with β-adrenergic/vasopressin-responsive bilateral macronodular adrenal disease with an ARMC5 mutation treated with metyrapone monotherapy for more than 5 years
Source: Front Endocrinol (Lausanne). 2026 Jul 21;17:1852080. doi: 10.3389/fendo.2026.1852080 (PMC13433298; doi:10.3389/fendo.2026.1852080)
Supplement: Supplementary file 1 [file Table1.docx]

| Supplemental Table 1. Endocrine evaluations |  |  |  |
| --- | --- | --- | --- |
| Hormonal measurements | Patient 1 |  | Patient 2 |
| Plasma ACTH (pg/mL)^a^ at 07:00 h | <1.0 |  | 8.4 |
| Plasma ACTH (pg/mL)^a^ at 23:00 h | <1.0 |  | 5.2 |
| Serum cortisol (μg/dL)^a^ at 7:00 h | 13.4 |  | 21.6 |
| Serum cortisol (μg/dL)^a^ at 23:00 h | 12.1 |  | 7.9 |
| Serum DHEA-S (ng/mL)^a^ | 604 |  | 72 |
| 24h urinary free cortisol (μg/day)^b^ | 94.5 |  | 69.4-101 |
| Plasma renin activity (ng/mL/hr)^c^ at rest | 2.1 |  | 1.5 |
| Plasma aldosterone concentration (pg/dL)^a^ at rest | 85.9 |  | 44.8 |
| Serum cortisol (μg/dL)^a^ in overnight 1 mg dexamethasone suppression test | 12.4 |  | 6.2 |
| Serum cortisol (μg/dL)^a^ in overnight 8 mg dexamethasone suppression test | 9.6 |  | 6.2 |
| Serum cortisol (μg/dL)^a^ in corticotropin-releasing hormone stimulation test | 14.8-21.5 (139%) |  | 13.7-33.8 (247%) |
| Plasma ACTH (pg/mL)^a^ in corticotropin-releasing hormone stimulation test | 1.4-9.2 (657%) |  | 1.6-59.9 (3740%) |
| Serum cortisol (μg/dL)^a^ in short-duration synthetic ACTH stimulation | 16.3-44.8 |  | 16.4-41.3 |

a, measured by CLEIA; b, measured by CLIA; c, measured by EIA

Normal reference ranges: serum cortisol at 08:00 h (7.07-19.6 μg/dL) and 24:00 h (< 5.0 μg/dL); plasma ACTH (7.2–63.3 pg/mL), DHEA-S (1060-4640 for Patient 1, 120-1330 for Patient 2), urinary free cortisol (4.3-176 μg/day), plasma renin activity at rest (0.2-2.3 ng/mL/hr), plasma aldosterone concentration at rest (4.0-82.1 pg/dL)
